# Removing Constraints on the Biomass Production of Freshwater Macroalgae by Manipulating Water Exchange to Manage Nutrient Flux

**DOI:** 10.1371/journal.pone.0101284

**Published:** 2014-07-07

**Authors:** Andrew J. Cole, Rocky de Nys, Nicholas A. Paul

**Affiliations:** MACRO — the Centre for Macroalgal Resources and Biotechnology, and School of Marine and Tropical Biology, James Cook University, Townsville, Queensland, Australia; Nanyang Technological University, Singapore

## Abstract

Freshwater macroalgae represent a largely overlooked group of phototrophic organisms that could play an important role within an industrial ecology context in both utilising waste nutrients and water and supplying biomass for animal feeds and renewable chemicals and fuels. This study used water from the intensive aquaculture of freshwater fish (Barramundi) to examine how the biomass production rate and protein content of the freshwater macroalga *Oedogonium* responds to increasing the flux of nutrients and carbon, by either increasing water exchange rates or through the addition of supplementary nitrogen and CO_2_. Biomass production rates were highest at low flow rates (0.1–1 vol.day^−1^) using raw pond water. The addition of CO_2_ to cultures increased biomass production rates by between 2 and 25% with this effect strongest at low water exchange rates. Paradoxically, the addition of nitrogen to cultures decreased productivity, especially at low water exchange rates. The optimal culture of *Oedogonium* occurred at flow rates of between 0.5–1 vol.day^−1^, where uptake rates peaked at 1.09 g.m^−2^.day^−1^ for nitrogen and 0.13 g.m^−2^.day^−1^ for phosphorous. At these flow rates *Oedogonium* biomass had uptake efficiencies of 75.2% for nitrogen and 22.1% for phosphorous. In this study a nitrogen flux of 1.45 g.m^−2^.day^−1^ and a phosphorous flux of 0.6 g.m^−2^.day^−1^ was the minimum required to maintain the growth of *Oedogonium* at 16–17 g DW.m^−2^.day^−1^ and a crude protein content of 25%. A simple model of minimum inputs shows that for every gram of dry weight biomass production (g DW.m^−2^.day^−1^), *Oedogonium* requires 0.09 g.m^−2^.day^−1^ of nitrogen and 0.04 g.m^−2^.day^−1^ of phosphorous to maintain growth without nutrient limitation whilst simultaneously maintaining a high-nutrient uptake rate and efficiency. As such the integrated culture of freshwater macroalgae with aquaculture for the purposes of nutrient recovery is a feasible solution for the bioremediation of wastewater and the supply of a protein resource.

## Introduction

The production of macroalgal biomass is a developing component of clean technologies for the remediation of wastewater and carbon dioxide within an integrated closed-loop cycle often referred to as industrial ecology [1], [2]. The ultimate aim of industrial ecology is to replicate the efficiencies observed in biological systems, where all ecosystem resources are recycled and the waste of one species becomes the food of another [1]. Using this framework, industrial ecology is primarily concerned with shifting industrial processes from linear systems, in which resources move through a system to become waste, to a closed-loop system where wastes become valued as an input for the next production process. A major part of this process is the integration of production systems so that waste products can be easily accessed either as a raw material or as an energy carrier, with the emphasis on processes and practices that reduce greenhouse gas emissions and related environmental impacts of waste streams [3]. On a global scale one of the largest and most consistent sources of industrial waste is nutrients – nitrogen and phosphorous. A large proportion of this waste is created as a by-product of intensive animal agriculture [4]. Globally, nitrogen and phosphorous waste now exceed 138 Tg.y^−1^ and 11 Tg.y^−1^ respectively [5]. These waste nutrients are currently seen as a liability and are either lost to the atmosphere through denitrification or leached into the local environment where nitrogen enrichment and eutrophication problems can occur with global scale impacts [6]–[12]. However, these excess nutrients need not be relegated to waste but rather could be recycled and utilised as an input resource for the large-scale cultivation of phototrophic organisms, themselves to be recycled again as bioproducts [13]–[15]. Inorganic nitrogen in particular is the primary limiting nutrient for the production of algae and as such this waste nitrogen could be an ideal resource for the large-scale production of algal biomass [16]. The cultivation and subsequent on-site use of macroalgal biomass at sites with high-nutrient waste streams, such as intensive livestock production, can close the loop between waste production, waste capture and re-use. The on-site use of cultured biomass has the additional benefit of reducing the energy and transportation costs associated with either bringing animal feeds to the farm or transporting algal biomass away.

Generally, the production of macroalgal biomass is highest when cultures are provided with a constant supply of nutrient-rich water [17]–[20]. In flow-through systems, nutrient supply (or nutrient flux as g nutrients.m^−2^.day^−1^) can be manipulated by either changing the nutrient concentration of the incoming water or by changing the rate of water exchange. This is particularly important for large-scale production facilities which, as a consequence of the large water volumes involved, will only be able to exchange relatively low amounts (e.g. 10–50%) each day. In contrast, the majority of small scale research to date has often used very high water exchange rates (>24 volumes per day) to obtain exceptionally high but commercially unobtainable rates of biomass production [20]–[22]. A high water exchange rate, in addition to supplying nutrients in excess, also benefits algal production by facilitating the removal of dead algal cells and limits the accumulation of bacteria and fouling organisms which can negatively impact productivity [19], [20]. Increasing the rate of water exchange can also benefit biomass production rates by increasing the supply and availability of dissolved inorganic carbon (DIC). Under intensive culture conditions the high density and photosynthetic activity of macroalgae can raise the pH and rapidly deplete the proportion of DIC that is available for photosynthesis [23], [24].

The majority of previous work on macroalgal production and the effects of changing nutrient flux have focused on marine species where productivity is positively influenced by increasing the rate of water exchange [18], [20]. However, it is currently unclear whether freshwater species conform to this paradigm. It is also unclear how low the rate of water exchange can be before its beneficial aspects are lost and whether a very low water exchange rate, e.g. 10% water exchange per day, supplemented with additional nutrients or any limiting trace elements, will stimulate biomass production as effectively as a higher water exchange rate of low-nutrient water. This includes carbon as a limiting factor in the growth of freshwater macroalgae [25].

The majority of research that has investigated the effect of changing nutrient flux on macroalgal productivity have focused on marine species [17], [18], [20], [21], [26], yet paradoxically most major industries with high-nutrient waste streams, such as intensive agriculture, mineral processing, energy production and municipal waste, are located around freshwater, rather than saltwater, environments. Our key target species for the bioremediation of freshwater waste streams are from the genus *Oedogonium*, a competitively dominant genus of filamentous macroalgae that also has a biochemical composition suitable for a range of biomass applications [25], [27], [28]. Therefore, this study investigates for the first time how the rate of biomass production of *Oedogonium* responds to both the rate of water exchange and the supplementary addition of nutrients and carbon when cultured using the wastewater from a freshwater aquaculture farm as a case study for the integration of freshwater macroalgal biomass for bioremediation and bio-products. This study also investigates the effect of these factors on the nitrogen content of freshwater macroalgae with the goal of understanding the relationship between productivity and protein content of *Oedogonium* in this system.

## Methods

### Ethics statement

Research on algae does not require ethics approval. All algae used in this study originated from stock cultures maintained at the Marine and Aquaculture Research Facility at James Cook University. This research complied with all Australian laws.

This study was undertaken on private land owned by Good Fortune Bay Fisheries LTD. Permission to use this land was granted by the farm manager, Rod Pelling. No other permission was needed to use this land or perform our experiment.

### Study species and site

*Oedogonium* is a genus of unbranched, uniseriate filamentous green algae made up of small cylindrical cells. This genus has a worldwide distribution and is a common component of natural ecosystems where it grows either attached to the substrate or as free floating mats. *Oedogonium* is a robust and competitively dominant genera that has been identified as a key target group for the bioremediation of freshwater waste streams [27] and as a feedstock biomass for bioenergy applications [25], [28], [29]. Stock cultures of *Oedogonium* sp. as described in Lawton et al. [27] (Genebank accession numbers: KC701472), and hereafter referred to as *Oedogonium*, were maintained at the Marine & Aquaculture Research Facilities Unit (MARFU), at James Cook University (JCU), Townsville (Latitude: 19.33°S; Longitude 146.76°E). The experimental component of this study was conducted at Good Fortune Bay Fisheries Ltd, a 450 tonne per annum freshwater barramundi farm located in Townsville, Australia. Barramundi are cultured in large outdoor ponds, constantly supplied with a supply of clean bore water. Pond effluent water is passively cleaned using reed beds and settlement ponds to reduce suspended solids before being released to the environment. In this experiment, pond effluent water was pumped out of the settlement pond and sand filtered to 150 µm. This water was then used to feed twenty cylindrical tanks (Duraplas AP 1000; 1000 L capacity, 1.19 m^2^ surface area) to a depth of 70 cm which gave a total culture volume of 853L. *Oedogonium* was maintained in tumble culture in these tanks through the use of a central aeration ring (45 cm circumference). A 750 µm mesh screen was used to prevent the *Oedogonium* filaments from exiting the tank with the outgoing water.

### Nutrient flux experiment

This experiment investigated the separate and additive effects of changing water exchange rates and the addition of nitrogen and carbon to cultures on the biomass production and nitrogen content of *Oedogonium*. Five water renewal rates of 0.1, 0.5, 1, 2.5 and 5 volumes per day were used, with these rates representing realistic water exchange rates that a large-scale algal production facility could use. Exchange rates were controlled using digital flow meters fitted to each tank (Hoselink digital flow meters). These flow meters dispensed an hourly allotment of water into each tank. The elemental composition of the settlement pond water that was used in this experiment was measured at the beginning and end of the experimental period, with elemental composition remaining consistent over this 6 week timeframe (Table 1). To investigate how the concentration of nitrogen and the availability of dissolved inorganic carbon (DIC) influences biomass production, the sand-filtered pond water was split across each water renewal rate into four treatments; pond water (PW), pond water plus nitrogen (PW+N), pond water plus carbon (PW+CO_2_) and pond water plus nitrogen and carbon (PW+N+CO_2_). In this study we used a randomised complete block design, which is an appropriate experimental design for longer term studies with uncontrolled variables that differ over time (e.g. temperature, light intensity), and replicates within each block are exposed to the same environmental conditions. This design enabled us to partition the effects of the factors of interest, water exchange rate, carbon addition and nitrogen addition, from any potential environmental effects. In our experiment we had two fixed water treatment factors with two levels of each factor, with and without carbon addition (C) and with and without nitrogen addition (N), and five water exchange (W) rates with five levels; 0.1, 0.5, 1, 2.5 and 5 volumes.day^−1^. In total, we had 20 replicate tanks and each of these tanks was assigned to 1 treatment combination (CxNxW) per replicate block. This experimental set up was replicated over five time periods (five blocks), giving a total of five replicates for each treatment combination.

**Table 1:** Elemental composition of fish pond water.

Metal	Concentration (mg.L^−1^)
Sodium	271–291
Calcium	47–59.4
Magnesium	47–128
Potassium	5.9–6.0
Strontium	0.55–0.73
Phosphate	0.13–0.50
Manganese	0.05–0.12
Iron	<0.05
Arsenic	<0.01
Selenium	<0.01
Vanadium	<0.02
Zinc	<0.02
Molybdenum	<0.005
Copper	<0.002
Nickel	<0.002
Aluminium	<0.001
Lead	<0.001
Cadmium	<0.0001
Mercury	<0.0001

Values are the ranges of two samples taken at the start and end of the experimental period.

Additional nitrogen was added to tanks by filling a 150 L conical tank with tap water and enriching this water with 850 g of ammonium chloride. A Kamoer 12 channel dosing pump was then used to supply a pulse addition of 120 mL of this ammonium-enriched water into each treatment tank every hour to provide an additional nitrogen flux of 0.2 mg.L^−1^.hr^−1^. The DIC concentration was increased through the dissolution of CO_2_ gas into pond water through a speece cone at a rate of 2.5 L.min^−1^. The CO_2_ supply was controlled via a digital timer and solenoid valve which limited carbon addition to the daylight hours between 8am and 4pm. A 150 mL sample of incoming pond water was collected on days one and three of this experiment to determine the ammonium, nitrite and nitrate concentrations for all treatments, while the total alkalinity was measured from the incoming water to both the PW and PW+CO_2_ treatments. Water samples were analysed by the Australian Centre for Tropical Freshwater Research at James Cook University. The pH and temperature of each tank was measured between 2–3pm every afternoon. The concentration of DIC and the proportion of each of the three carbon species available (CO_2_, HCO_3_^−^ or CO_3_^2−^) in the culture water were calculated using the software CO_2_sys [30] based on the total alkalinity of the incoming water and the pH and temperature of each replicate culture. This experiment was conducted over a 6 week period between 6^th^ May and 14^th^ June 2013. During this period the daily water temperature of the tanks ranged between 18.9 and 24.3°C, the mean daily Photosynthetic Active Radiation was 23.2 (±1.5) mol photons.m^−2^, (range:14.8–31.2 mol.m^−2^) with daily peaks ranging between 1034 and 1914 µmol photons.m^−2^.s^−1^, and the nitrogen and phosphorous concentration of the pond water ranged between 1.93–2.75 mg.L^−1^ and 0.16–0.53 mg.L^−1^ respectively.

*Oedogonium* was stocked in each tank at a density of 0.35 g.L^−1^ (300 g fresh weight: FW). The algae used to stock these tanks had been continuously cultured for three weeks prior to this experiment using sand filtered pond water at a water renewal rate of 1 tank volume per day (1 vol.day^−1^). The biomass used to stock the tanks had a mean internal nitrogen content of 5.04 (±0.4) %. To quantify productivity, algal biomass was harvested after 4 days, centrifuged using a domestic washing machine (1000 rpm) to remove excess water, weighed and dried at 60°C for 48 hours before being reweighed to provide dry weight (DW). Algal productivity was calculated using the equation: 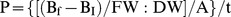, where *B_f_* and *B_I_* are the final and initial algal biomass, *FW:DW* is the fresh to dry weight ratio, *A* is the area of culture tanks and *t* is the number of days in culture. Separate mixed-model ANOVAs were used to test for differences in biomass production rates, internal nitrogen content and maximum daily pH of cultures with water exchange rates (n = 5 treatments) and incoming treatment water (with and without nitrogen; with and without carbon) as our fixed factors, while time (growth trial) was treated as the blocking factor. Residual plots were examined to ensure ANOVA assumptions were met. Tukey's multiple comparison tests were then used to compare the means of treatment groups.

One gram of dried biomass was then sent to OEA Laboratories UK to determine the internal nitrogen content, while the remaining 1 g was sent to the Advanced Analytical Centre at JCU to determine the total phosphorous content of the biomass. The internal nitrogen and phosphorous concentrations were then used to calculate the uptake rate and uptake efficiencies of the cultures. Uptake rate was calculated in g.m^−2^.day^−1^ and was the difference between the initial and final concentrations in the biomass divided by the culture period (4 days) and culture surface area. Uptake efficiency was calculated in % and was the proportion of nitrogen and phosphorous in incoming water that was incorporated into the biomass. Protein content of the *Oedogonium* biomass was calculated by multiplying the nitrogen content by 4.7 following Neveux et al. [28], which is a conservative conversion as it does not include the amino acids Cysteine and Tryptophan [28].

## Results

**Figure 1:**
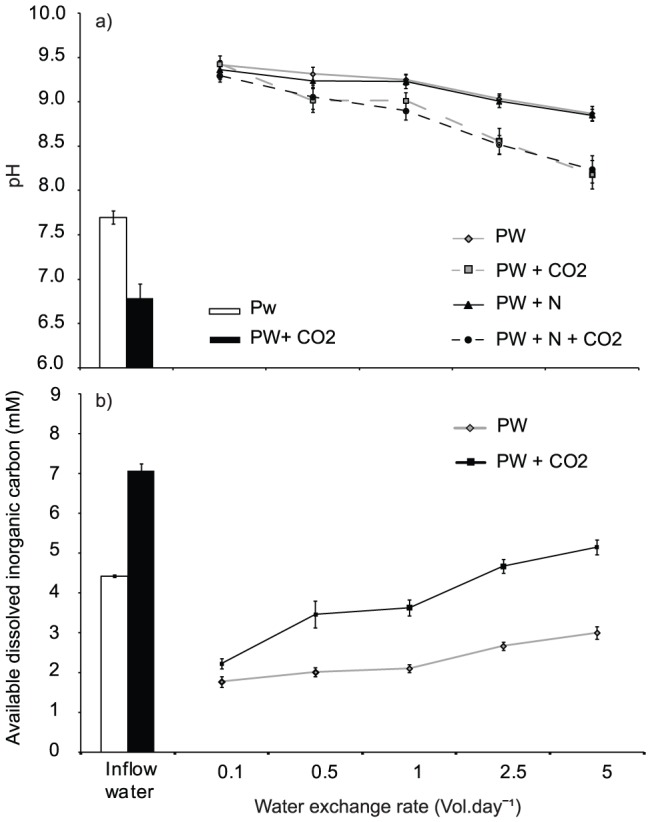
pH and carbon availability of culture water. a) Average maximum daily pH and b) total dissolved inorganic carbon available (CO_2_ and HCO_3_^−^) to *Oedogonium* cultures at five water exchange rates (0.1, 0.5, 1, 2.5 and 5 vol.day^−1^) using fish pond water with and without nitrogen (N) and carbon (CO_2_) supplementation. In graph b nitrogen treatments have been excluded as nitrogen had no significant effect on carbon availability.

The average (± SE) pH of inflow water was 7.69 (±0.07) and 6.78 (±0.16) for PW and PW+CO_2_ treatment water respectively. The addition of CO_2_ had only a minor effect on the pH of the low flow treatments with the average daily maximum pH of these treatments ranging between 9.29 and 9.42 (Fig. 1b). The addition of nitrogen to pond water had no significant effect on maximum daily pH values within each tank. However, the pH of cultures was significantly affected by CO_2_ addition (P<0.001) and water exchange rate (P<0.001) (Table 2). The pH decreased with both CO_2_ addition and increasing rates of water exchange. As the rate of water exchange increased the effect of carbon addition on pH became more pronounced with a difference of 0.65 pH units between tanks with (∼8.21) and without (∼8.86) carbon supplementation at a water exchange rate of 5 vol.day^−1^ (Fig. 1a). This interaction between CO_2_ addition and water exchange was significant (P = 0.002) (Fig. 1b).This difference in pH resulted in a 25% increase in the proportion of DIC available to cultures supplemented with CO_2_ in the lowest water exchange rates and an increase of 70–75% for water exchange rates between 0.5–5 vol.day^−1^ (Fig. 1b).

**Table 2:** Mixed model ANOVA results testing the effect of water exchange rates (0.1, 0.5, 1, 2.5 and 5 vol.day^−1^) and water treatment (Pond water with and without nitrogen and CO_2_ addition) on the pH of culture water over five replicate trials.

Source	Effect	DF	MS	F	P
Water exchange rate (W)	Fixed	4	1.55	70	<0.001
Nitrogen (N)	Fixed	1	0.08	3.4	0.07
Carbon (C)	Fixed	1	3.32	149.6	<0.001
W X N	Fixed	4	0.04	1.6	0.18
W x C	Fixed	4	0.1	4.6	0.002
N X C	Fixed	4	0.01	0.5	0.48
W x N x C	Fixed	4	0.03	1.1	0.35
Trial (T)	Random	4	0.04	1.6	0.18
Error		76	0.02		

**Figure 2:**
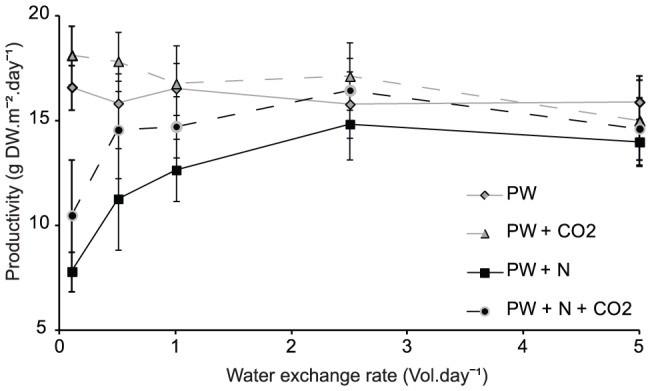
*Oedogonium* productivity. Biomass production rates of *Oedogonium* cultured at 5 water exchange rates, 0.1, 0.5, 1, 2.5 and 5 vol.day^−1^, using fish pond water (PW) with and without nitrogen (N) and carbon (CO_2_) supplementation. Values are the means and SE of 5 replicate growth trials.

*Oedogonium* productivity ranged between 3.8 and 23.8 g DW.m^−2^.day^−1^ for all treatments combined, although 76% of these replicate tanks had productivities greater than 12 g DW.m^−2^.day^−1^. Biomass production was significantly influenced by the addition of nitrogen (P<0.001) and carbon (P = 0.002) and there was a significant interaction between nitrogen addition and the rate of water exchange (P = <0.001) (Table 3). This interaction was driven primarily by the negative response of *Oedogonium* to nitrogen supplementation at low flow rates (Fig. 2). At low water exchange rates the addition of nitrogen resulted in the lowest growth rates with mean productivities of 7.8 (±0.9) and 10.5 (±2.6) g DW.m^−2^.day^−1^ in the PW+N and PW+N+CO_2_ treatments, respectively. As the rate of water exchange increased the productivity of the nitrogen addition treatments increased and peaked at a water exchange rate of 2.5 vol.day^−1^. This combination had a mean productivity of 14.8 (±1.7) g DW.m^−2^.day^−1^ in the PW+N treatment and 16.5 (±1.5) g DW.m^−2^.day^−1^ and PW+N+CO_2_ treatment. In contrast, at the lowest water exchange rates *Oedogonium* cultured in raw pond water had the highest productivities. A 10% daily water exchange resulted in a mean productivity of 16.6 (±1.1) g DW.m^−2^.day^−1^ in pond water and 18.1 (±1.4) g DW.m^−2^.day^−1^ when CO_2_ was added to incoming pond water. Increasing water exchange rates in these treatments resulted in a steady 5–15% decline in productivity with the highest water exchange treatment of 5 vol.day^−1^ having mean productivities of 15 (±2.1) g DW.m^−2^.day^−1^ for PW and 15.9 (±1.1) g DW.m^−2^.day^−1^ for PW + CO_2_ treatments. The addition of CO_2_ to cultures generally had a positive effect on productivity and resulted in up to a 25% increase in productivity at the lowest water exchange rates (Fig. 2).

**Table 3:** Mixed model ANOVA results testing the effect of water exchange rates (0.1, 0.5, 1, 2.5 and 5 vol.day^−1^) and water treatment (Pond water with and without nitrogen and CO_2_ addition) on the biomass productivity of *Oedogonium* cultured over five replicate trials.

	Effect	DF	MS	F	P
Water exchange rate (W)	Fixed	4	0.04	0.28	0.60
Nitrogen (N)	Fixed	1	42.26	54.72	<0.001
Carbon (C)	Fixed	1	2.63	9.55	0.002
W X N	Fixed	4	8.28	11.87	<0.001
W x C	Fixed	4	4.13	1.82	0.13
N X C	Fixed	1	7.09	1.49	0.23
W x N x C	Fixed	4	0.06	0.10	0.98
Trial (T)	Random	4	0.32	2.43	0.06
Error		76	0.02		

**Figure 3:**
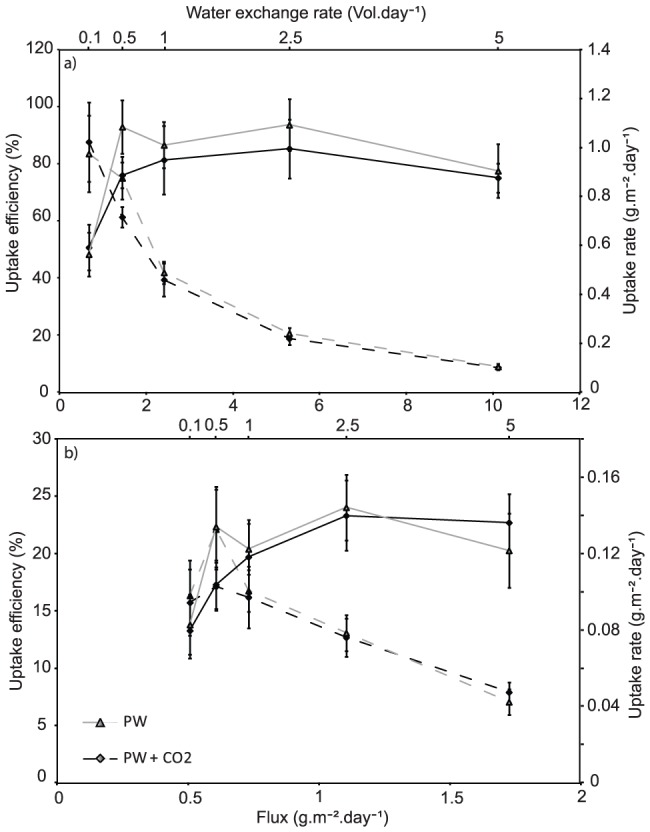
Nitrogen and phosphorous uptake rates and efficiencies. Uptake rates (solid lines) and efficiency (dashed lines) of *Oedogonium* when cultured in pond water (grey lines) and pond water +CO_2_ (black lines) under increasing nutrient flux of a) nitrogen and b) phosphorous. For reference purposes the five water exchange rates that these nutrient fluxes correspond to have been added to the top x-axis.

Settlement pond effluent had a total dissolved inorganic nitrogen content of 2.43 (±1.39) mg.L^−1^ and a filterable reactive phosphorous concentration of 0.34 (±0.02) mg.L^−1^. The total nitrogen concentration was made up of 1.06 (±0.16) mg.L^−1^ as total ammonia nitrogen (TAN), 0.47 (±0.02) mg.L^−1^ as nitrite-N and 1.35 (±0.18) mg.L^−1^ as nitrate-N. *Oedogonium* cultured in this water had a total nitrogen uptake rate that ranged between 0.45 (±0.09) and 1.09 (±0.11) g.m^−2^.day^−1^ (Fig. 3a) and a mean phosphorous uptake rate ranging between 0.08 (±0.01) and 0.15 (±0.02) g.m^−2^.day^−1^ (Fig. 3b), with the lowest uptake rates occurring in the 0.1 vol.day^−1^ treatments for both nutrients. At the asymptote point of uptake rates, which corresponded to a nitrogen and phosphorous flux of 1.5 g.m^−2^.day^−1^ and 0.75 g.m^−2^.day^−1^ respectively, uptake efficiencies of 61 (±3.6) and 75.2 (±7.5) % for nitrogen and 15.7 (±2.9) and 22.1 (±3.5) % for phosphorous were recorded in the PW and PW+CO_2_ treatments, respectively. Further increases in nitrogen and phosphorous fluxes did not increase the uptake rate and subsequently decreased the uptake efficiency of nitrogen and phosphorous to lows of 8 and 7% for the highest water exchange treatment which corresponded to a nitrogen and phosphorous flux of 10.1 and 1.7 g.m^−2^.day^−1^ respectively (Fig. 3).

**Figure 4:**
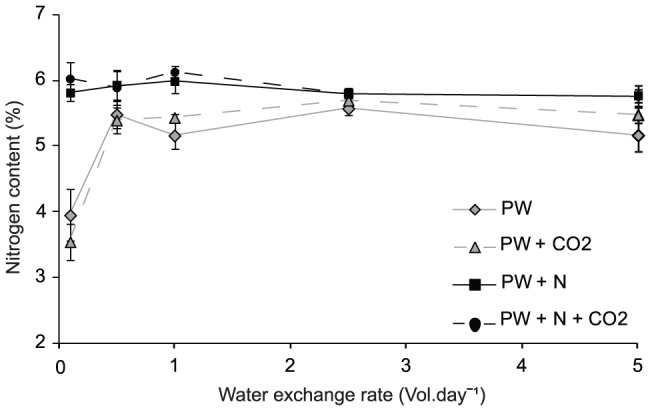
*Oedogonium* nitrogen content. Mean (±SE) internal nitrogen content of *Oedogonium* cultured under 5 water exchange rates (0.1, 0.5, 1, 2.5 and 5 vol.day^−1^) using fish pond water (PW) with and without nitrogen (N) and carbon (CO_2_) supplementation. Note protein content of *Oedogonium* can be calculated by multiplying %N by 4.7 [28].

The internal nitrogen content of the *Oedogonium* biomass ranged from 2.5–6.7% across all treatments (Fig. 4). The cultures with the highest productivities, cultured at the lowest water exchange rate, had the lowest internal nitrogen content with a mean nitrogen content of 3.9 (±0.39) % for PW and 3.54 (±0.28) % for PW+CO_2_ treatments. Increasing the water exchange rate from 0.1 to 0.5 vol.day^−1^ resulted in a 38% and 53% increase in the internal nitrogen content of the *Oedogonium* biomass cultured in the PW and PW+CO_2_ treatments respectively. Water exchange rates from 0.5–5 vol.day^−1^ resulted in an internal nitrogen content ranging from 4.94–6.26%, with the mean internal nitrogen content peaking at 5.57 (±0.11) % and 5.69 (±0.06) % at a water exchange rate of 2.5 vol.day^−1^for the PW and PW+CO_2_ treatments respectively (Fig. 3). These nitrogen values translate to a crude protein content, using the 4.7 nitrogen conversion factor, ranging from 16.7 (±1.3) %, at the lowest water exchange rate, to a high of 26.7 (±0.3) %, at a water exchange of 2.5 vol.day^−1^when cultured using PW and PW+CO_2_ water. In the nitrogen addition treatments the internal nitrogen content was relatively stable and ranged between 5.19 and 6.71% with almost two-thirds of the cultures having an internal nitrogen content greater than 5.9%. In these treatments the mean internal nitrogen content peaked at a water exchange rate of 1 vol.day^−1^ with an average nitrogen content of 5.99 (±0.18) % and 6.13 (±0.08) % for PW+N and PW+N+CO_2_ water respectively. The protein content of *Oedogonium* cultured in the PW+N treatments was largely independent of flow rates and ranged between 26.0 (±2.7) % and 28.8 (±0.4) %.

## Discussion

This study has demonstrated that freshwater macroalgae can be cultured using the nutrient wastewater from a freshwater fish farm. We expect that the cultures of *Oedogonium* could be successfully integrated with any industry or process that produces large quantities of similar wastewater. This study has also demonstrated that *Oedogonium* can be successfully cultured in a wide range of nutrient concentrations, is amenable to relatively low water exchange rates and is an extremely robust species that can maintain pure cultures in open systems under field conditions; a problem that severely impacts the scale up of microalgal cultures [31]–[33]. The biomass production rates in our study were comparable to other studies on freshwater macroalgae, which range between 5–25 g.m^−2^.day^−1^ [15], [16], [34]–[37]. These are higher than the 8 g.m^−2^.day^−1^ biomass production rates for microalgae cultured in outdoor ponds using raw industry waste water [38]. Encouragingly, this study demonstrates that there is no need for high flow rates to maintain the production of *Oedogonium* and that a flow rate between 0.5 and 1 vol.day^−1^ is sufficient to maintain high biomass productivity and therefore high uptake efficiency of nitrogen and phosphorous. Additionally the biomass properties of *Oedogonium*, particularly its crude protein content, can be manipulated by controlling the amount of nitrogen the cultures receive, either through changing water renewal rates or through the addition of a concentrated nitrogen waste stream. Culturing *Oedogonium* at flow rates of 0.1 vol.day^−1^, where nitrogen availability is reduced, resulted in a biomass that was highly productive but had a relatively low protein content of 16% (related to a low internal nitrogen content of 3.5%). This biomass is well suited to bioenergy applications, specifically the conversion to liquid fuels through hydrothermal liquefaction [28], [39], [40]. Likewise the *Oedogonium* biomass produced at higher water exchange rates, 0.5–1 vol.day^−1^, where nitrogen was not limited, resulted in a biomass that had a high protein content (26–28%), which could makes this biomass an ideal feed supplement or replacement option for livestock. Several species of algae have already been successfully incorporated into the diets of livestock, with algae generally having positive effects on animal health, productivity and product quality [41]–[46].

Worldwide, protein used in the diets of animals exceeds 150 million tonnes annually, with soybean meal accounting for the bulk of this protein [47]. However, the trend of increasing population sizes and the increasing proportion of meat in the diets of developing countries means that in the future unconventional sources of protein will be required to meet global protein needs [47], [48]. The crude protein content of *Oedogonium* in this study is lower than that of most species of microalgae (20–60%) [49], [50], but compares well to other species of macroalgae (20–40%) [28], [34], [51], [52]. *Oedogonium* also has a comparable protein content to conventional sources of protein such as whole soybeans at 32–43% [52]–[55] and is higher than most cereals and grains which range between 8–16% [54], [56]–[59]. Even relatively novel terrestrial sources of crude protein such as Kenaf (*Hibiscus cannabinus*) or *Moringa oleifera* have a leaf protein content ranging between 15–26% [60]–[62], however when the entire plant biomass is taken into account the total crude protein content is much lower than the 16–28% of *Oedogonium*. In this regard, freshwater macroalgae could be an ideal alternative protein source to feed to livestock, especially if this biomass is cultured using farm waste nutrients and re-used on the same site [34], [51], [63]. We do not expect the cultured *Oedogonium* biomass to have any animal feed restrictions related to heavy metal accumulation as the water used to culture this biomass is from a freshwater source that is only used for animal production (aquaculture) and has no exposure to anthropogenic contaminants. Further, the same species of *Oedogonium* has previously been cultured in ash dam water (high in contaminants) and the resulting biosolid concentrations of these contaminants were not high compared to seaweeds which are currently used in numerous biomass applications [29], [64].

The ability of *Oedogonium* to adapt and be successfully cultured in a wide range of flow rates makes it particularly suited to larger, industrial-scale cultivation, where large quantities of wastewater can be cleaned whilst simultaneously producing a feedstock biomass that can be tailored to meet different biomass applications. At these larger scales, moving large volumes of water becomes prohibitively expensive. This is one of the limitations of marine macroalgae (seaweed), where relatively high water exchange rates, greater than 12 volumes per day are required to maximise biomass production rates [20], [21]. These high water exchange rates provide a constant supply of nitrogen but also prevent carbon limitation as the pH of culture water is kept relatively low [20], [23], [24]. In contrast, *Oedogonium* had its highest productivity at the lower water exchange rates 0.1–0.5 vol.day^−1^, where nitrogen availability was reduced. At the lowest water exchange rates, the *Oedogonium* biomass was partly utilizing its internal nitrogen reserves to generate new biomass; the internal nitrogen content of these cultures decreased by 22–30% during the culture period. This indicates that processing inorganic nitrogen from the water column is potentially an energetically expensive process relative to using nitrogen that has already been processed and stored within the cell. We expect that this feature can be used to our advantage and that managing cultures with periodic nitrogen limitation followed by pulse additions of nitrogen could result in further increases in biomass production rates. These periodic water renewals would also have the additional benefit of increasing the energy efficiency of the culture system as pumping costs could be further reduced.

While the addition of CO_2_ to incoming water increased productivity, this increase (between 2 and 25%) was much lower than the 2.5 fold increase in productivity previously identified when *Oedogonium* was cultured in batch conditions with weekly water exchanges [25]. The main difference between these studies is the use of bore water in the current study, which had a total alkalinity ranging between 210–227 mgCO_3_.L^−1^ compared to 40–60 mgCO_3_.L^−1^ for the dechlorinated town water which was used in Cole et al. [25]. This higher alkalinity means that for the same pH and temperature, the culture water in the current study had a DIC concentration four times higher than when using dechlorinated townwater. This is an important result as it demonstrates that macroalgae can be successfully cultured at high productivities in areas which do not have access to a source of waste CO_2_, provided that the alkalinity of the culture water can meet the carbon needs of the algae. This may be a particularly useful approach if *Oedogonium* is used as an industrial tool for carbon capture and offset, whereby each dry weight kilogram of cultured biomass extracts ∼400 g of carbon from the water [25].

An unexpected result from this study was the decrease in productivity when pond water was supplemented with nitrogen, especially at low water exchange rates. A consequence of the way nitrogen was added to our tanks is that the ammonium accumulates in the low exchange rate treatments as each hourly addition increases the ammonium concentration until a new equilibrium is reached between the concentration of the ammonium-addition water and the dilution effect of the incoming pond water. In our lowest exchange rates the TAN concentration continually increased over the culture period up to 17 mg.L^−1^ which was four and nine times higher than the tanks receiving a water exchange rate of 0.5 and 1 vol.day^−1^ respectively. The decrease in productivity observed in these cultures is most likely a result of ammonium toxicity. In solution, ammonia occurs in two forms, ammonium (NH^4+^) and unionised ammonia (NH_3_). NH_3_ is toxic to animals and plants, as it is uncharged, lipid soluble and can traverse biological membranes [65]. The proportion of each form is primarily dependent upon pH, with the proportion of NH_3_ increasing rapidly as the pH exceeds 9 [66], [67]. At a water temperature of 22°C the proportion of NH_3_ increases from 12.7% at a pH of 8.5 to 31.5% at pH 9, 59.2% at pH 9.5 and 82.1% at pH 10 [66]. The pH of the low flow treatments (10% exchange) ranged between 9.1–9.36. Subsequently, these low water exchange treatments had a 58% lower productivity than raw pond water, however, as the rate of water exchange increased and the pH of culture water decreased this difference in productivity between treatments converged. The importance of reducing the pH to below 9 can also be seen in our carbon addition treatment, where at exchange rates greater than 0.5 vol.day^−1^ the additional carbon could maintain the pH below 9 and productivity was not significantly impaired. In contrast, in treatments without the addition of CO_2_ to reduce culture pH, ammonium toxicity decreased productivity until flow rates reached 2.5 volumes per day. A similar effect of ammonium toxicity was observed for duckweed, where productivity decreased linearly as the proportion of NH_3_ increased and the maximum tolerance level for unionised ammonia was approximately 8 mg NH_3_-N.L^−1^ [65]. The ability of *Oedogonium* to survive relatively high TAN concentrations with only sublethal effects on growth is a positive result and indicates that *Oedogonium* would be a suitable species to remediate sewage wastewater, which can have TAN concentrations exceeding 20 mg.L^−1^ [38], [68], as long as a carbon source is supplied to limit the rise in culture pH and the resultant toxicity of NH_3_ [65].

## Conclusion

This study has demonstrated that *Oedogonium* can be successfully cultured at low water exchange rates using fish farm discharge water without any need for nutrient or carbon supplementation. We have also demonstrated that the optimal culture of *Oedogonium* occurs at relatively low exchange rates of between 0.5–1 vol.day^−1^ and does not require high concentrations of nitrogen and phosphorous to grow at high productivities. Production rates were generally highest when nutrients were supplied in only slight excess to the theoretical amount required to maintain growth at any given internal nitrogen content. In this study a nitrogen flux of 1.4 g.m^−2^.day^−1^, and a phosphorous flux of 0.6 g.m^−2^.day^−1^ was the minimum needed to maintain *Oedogonium* growth at 16–17 g DW.m^−2^.day^−1^ and a protein content of 25%. A simple model of minimum inputs shows that for every gram of productivity (g DW.m^−2^.day^−1^), *Oedogonium* requires 0.09 g.m^−2^.day^−1^ of nitrogen and 0.04 g.m^−2^.day^−1^ of phosphorous to maintain growth without nutrient limitation whilst simultaneously maintaining a high-nutrient uptake rate and efficiency. In this regard the culture of *Oedogonium* on farms for the purpose of nutrient recovery is an effective bioremediation solution for treating nutrient wastewater while also supplying a useful protein resource to the agricultural industry.
